# Physiological Correlates of Endurance Time Variability during Constant-Workrate Cycling Exercise in Patients with COPD

**DOI:** 10.1371/journal.pone.0017007

**Published:** 2011-02-28

**Authors:** Isabelle Vivodtzev, Philippe Gagnon, Véronique Pepin, Didier Saey, Louis Laviolette, Cynthia Brouillard, François Maltais

**Affiliations:** 1 Centre de Recherche, Institut Universitaire de Cardiologie et de Pneumologie de Québec, Université Laval, Québec, Canada; 2 Inserm ERI17, HP2-REx-S Laboratory, Centre Hospitalier Universitaire de Grenoble, Grenoble, France; 3 Centre de Recherche, Hôpital Sacré-Coeur and Department of Exercise Science, Concordia University, Montréal, Québec, Canada; 4 Centre de Recherche, Groupe Hospitalier Pitié-Salpêtrière, Paris, France; Pennington Biomedical Research Center, United States of America

## Abstract

**Rationale:**

The endurance time (T_end_) during constant-workrate cycling exercise (CET) is highly variable in COPD. We investigated pulmonary and physiological variables that may contribute to these variations in T_end_.

**Methods:**

Ninety-two patients with COPD completed a CET performed at 80% of peak workrate capacity (W_peak_). Patients were divided into tertiles of T_end_ [Group 1: <4 min; Group 2: 4–6 min; Group 3: >6 min]. Disease severity (FEV_1_), aerobic fitness (W_peak_, peak oxygen consumption [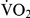
_peak_], ventilatory threshold [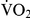
_VT_]), quadriceps strength (MVC), symptom scores at the end of CET and exercise intensity during CET (heart rate at the end of CET to heart rate at peak incremental exercise ratio [HR_CET_/HR_peak_]) were analyzed as potential variables influencing T_end_.

**Results:**

W_peak_, 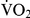
_peak_, 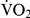
_VT_, MVC, leg fatigue at end of CET, and HR_CET_/HR_peak_ were lower in group 1 than in group 2 or 3 (p≤0.05). 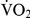
_VT_ and leg fatigue at end of CET independently predicted T_end_ in multiple regression analysis (r = 0.50, p = 0.001).

**Conclusion:**

T_end_ was independently related to the aerobic fitness and to tolerance to leg fatigue at the end of exercise. A large fraction of the variability in T_end_ was not explained by the physiological parameters assessed in the present study. Individualization of exercise intensity during CET should help in reducing variations in T_end_ among patients with COPD.

## Introduction

Exercise intolerance is a major concern in patients with chronic obstructive pulmonary disease (COPD) because it is related to poor prognosis and impaired quality of life [1], [2], [3]. Constant-workrate cycling or walking protocols are frequently used to assess the impact of COPD on functional capacity and the response to therapy [1]. The ability of these exercise protocols to detect improvements in exercise tolerance after various interventions such as bronchodilation [4], [5], [6], [7], [8], [9] or pulmonary rehabilitation [10], [11] has been demonstrated. One limitation of constant-workrate exercise tests (CET) is the large variation in endurance time among subjects, despite the fact these protocols are performed, by definition, at the same relative workload [9], [12], [13]. Since exercise duration during CET is frequently used as an outcome parameter in COPD clinical trials, the large interindividual variations in endurance time is highly relevant because it markedly influences sample size calculation. Also, because of the hyperbolic nature of the power-duration relationship of a constant workrate exercise test, the magnitude of improvement in endurance time following a given intervention will be influenced by its baseline pre-treatment value: the longer the pre-treatment endurance time, the larger the post-intervention improvement [14]. This obviously complicates the interpretation of improvements that are seen after an intervention. Being able to reduce this interindividual variability in endurance time at baseline would allow the design of smaller and less costly clinical trials whose results would also be easier to interpret.

The multifactorial nature of exercise intolerance in COPD may explain, in part, such variability in endurance time to CET. In a previous study, higher breathlessness scores were associated with reductions in endurance time in patients with COPD [15]. However, the potential role of extrapulmonary factors, such as physical fitness and the ability to tolerate exercise-related symptoms (e.g., dyspnea and leg discomfort), as contributors to the variability in endurance time has not been investigated. Technical factors could also play a role; for example underestimation or overestimating peak workrate capacity would lead to the performance of a CET at an intensity not truly representing 80% of peak capacity.

From a physiological standpoint, the power-duration relationship, characterizing the time for which a given exercise intensity can be sustained, has been proposed to determine the optimal workload in endurance tests [14], [15]. However, determining this relationship individually is not clinically practical because it requires the completion of several exercise tests of various intensities and durations. We therefore wished to address the issue of variability in endurance time from a clinical perspective with the idea that the identification of pulmonary function and exercise physiology parameters closely linked to endurance time could eventually be useful to help predict and reduce heterogeneity in the duration of CET.

In order to address the issue of variability in endurance time, we *a priori* posited the following hypotheses: individual variations in 1) disease severity, 2) aerobic fitness (peak exercise capacity, ventilatory threshold) and quadriceps strength, 3) the tolerance to dyspnea and leg fatigue during exercise, and 4) the actual (as opposed to estimated) exercise intensity during CET, could all contribute to influence exercise duration during a constant-workrate cycling exercise in patients with COPD. In this study, we tested these hypotheses by evaluating the variations in endurance time in 92 patients with COPD that performed a constant-workrate cycling exercise test at 80% of peak exercise capacity.

## Materials and Methods

### Patients

Ninety-two consecutive patients with stable COPD that performed a constant-workrate cycling exercise test (CET) in our laboratory between December 2001 and December 2004 were included in this study. Patients with COPD [forced expiratory volume in 1 s (FEV_1_) <80% predicted and FEV_1_/forced vital capacity (FVC) <0.7] had moderate to severe airflow obstruction [16]. Subjects were excluded of the study if they presented any medical conditions potentially influencing exercise testing (i.e., clinically diagnosed cardiovascular diseases [except hypertension], beta-blockers therapy, anemia, neurological, other respiratory disorders than COPD, recent exacerbation [<4 wk]).

### Ethics statement

The research protocol was approved by the institutional ethics committee (Research ethics committee of the *Institut universitaire de cardiologie et de pneumologie de Québec*) and a signed informed consent was obtained from each subject.

### Study design

After pulmonary function measurements, all patients performed a symptom-limited incremental cycle exercise to determine peak workrate capacity (W_peak_). On a separate day, quadriceps strength was measured before the performance of a CET at an intensity of 80% W_peak_. To evaluate the degree of muscle fatigue occurring during CET, the strength of the quadriceps was reassessed after CET. Spirometry was done before CET to ensure that patients were in stable condition. Patients were instructed to take their regular inhalation therapy during the study.

### Pulmonary function tests

Standard pulmonary function tests including spirometry, lung volumes and DL_CO_ were obtained according to previously described guidelines [17] and related to normal predicted values from the European Community for Coal and Steel/European Respiratory Society [18].

### Incremental Exercise Test

A symptom-limited progressive exercise test was performed on a cycloergometer (Quinton Corival 400; A-H Robins, Seattle, WA). Gas exchanges parameters (oxygen uptake, [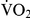], CO_2_ excretion [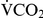], minute ventilation [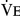]) and heart rate [HR] were measured monitored by a breath-by-breath exercise circuit (Sensor Medics, Vmax Legacy, Yorba Linda, CA). After 5 minutes of rest, the workload was initially set at 10 watts and subsequently increased at 1-minute intervals by 10-watt increments until the patient reached a symptom-limited maximum. W_peak_ and peak oxygen consumption (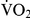
_peak_) were used as the index of peak exercise capacity. The ventilatory threshold was assessed using the V-Slope method [1] and reported as the 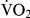
at the inflection point of the 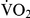/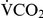 relationship (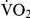
_VT_). Maximum voluntary ventilation (MVV) was estimated by multiplying FEV_1_ by 35 [19]. Ventilatory reserve was estimated from the 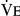/MVV ratio.

### Constant-workrate exercise test (CET)

After 2 min of unloaded pedaling, the workrate was set at 80% W_peak_ while patients were pedaling at 60–70 rotations per minute, up to exhaustion. No encouragement was provided during the tests to avoid any potential confounding effect on exercise performance [20]. The exercise test was stopped when the patient was unable to maintain a pedalling rate of 40 rpm. The endurance time was defined as the duration of CET excluding the 2-min warm-up period. Inspiratory capacity (IC) and pulse oxygen saturation (SpO_2_) were measured at rest and at the end of exercise.

### Quadriceps strength and fatigue

In seventy-six patients, the strength of the quadriceps was assessed during volitional maximal contraction (MVC) and during magnetic stimulation of the femoral nerve (quadriceps twitch force [Twq]) as described by Saey and colleagues [21]. Twq was also measured 10 min after CET to assess the post-exercise fall in quadriceps strength. Contractile quadriceps fatigue was defined as a post-exercise reduction in Twq of more than 15% from the resting value [21].

### Subjective Measures

Dyspnea and perception of leg fatigue were evaluated at end of incremental exercise and CET, using the modified 10-point Borg scale [22].

### Data analyses

Variables potentially influencing endurance time were grouped into 4 categories: *i)* disease severity, *ii)* aerobic fitness and quadriceps function, *iii)* tolerance to exercise dyspnea and leg fatigue *iv)* technical factors. These variables are presented in Table 1.

**Table 1:** Variables potentially influencing endurance time.

Categories	Variables
**Disease severity**	**Airflow limitation** : FEV_1_ **Hyperinflation** : TLC, FRC, RV and IC
**Aerobic fitness and quadriceps function**	**Fitness** : W_peak_ , 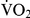 _peak_ , 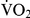 _VT_ **Quadriceps strength**: MVC and Twq**Quadriceps fatigue** : fall in Twq after CET
**Tolerance to dyspnea and leg fatigue**	**Dyspnea** and **leg fatigue** at end of CET
**Technical factors**	**Optimality of incremental exercise test:**HR_peak_ (% predicted value) 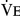 /MVVRespiratory exchange ratio**Actual intensity of CET:**HR_CET_/HR_peak_ 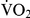 _CET_/ 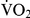 _peak_ 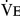 _CET_/ 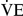 _peak_

*Definitions of abbreviations: CET: constant-workrate exercise test; HR: heart rate;* 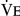 *: minute ventilation; MVV: maximal voluntary ventilation; FEV_1_: forced expiratory volume in 1 second; TLC: total lung capacity; FRC: functional residual capacity; RV: residual volume; IC: inspiratory capacity;* 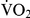 *: oxygen consumption;* 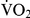 *_VT_: ventilatory threshold; MVC: maximal voluntary contraction.*

#### Disease severity

We speculated that worse airflow limitation, as assessed by FEV_1_ and hyperinflation (total lung capacity [TLC], functional residual capacity [FRC], residual volume [RV] and inspiratory capacity [IC]) would be associated with lower endurance time.

#### Aerobic fitness and quadriceps function

We hypothesized that a lower fitness level (W_peak_, 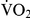
_peak_, 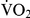
at the ventilatory threshold [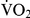
_VT_]) would be associated with a reduced endurance time. We lastly speculated that weaker quadriceps and a large post-exercise fall in TwQ at 10 minutes post CET would be associated with a reduced endurance time. This hypothesis was based on the observation that the susceptibility to muscle fatigue may contribute to exercise intolerance in COPD [21], [23].

#### Tolerance to dyspnea and leg fatigue

We posited that the endurance time would be influenced by the tolerance to exercise-related symptoms. Our hypothesis was that lower dyspnea and leg fatigue scores at the end of CET, representing poor tolerance to symptoms, would be associated with a reduced endurance time. The rationale for this hypothesis was that fitter individuals usually reach higher symptom scores during exercise compared to individuals less accustomed to the uncomfortable sensations of exercise [24].

#### Technical factors

We speculated that underestimating W_peak_ would result in CET being performed below the desired intensity (80% true peak capacity) and, as a result, in a prolonged endurance time. Peak heart rate (HR_peak_), expressed as a percentage of the predicted value (220-age) [1], 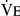/MVV and respiratory exchange ratio were used to characterize the intensity of incremental exercise test. The actual intensity of CET was evaluated by calculating the ratio of heart rate at the end of CET to heart rate at peak incremental exercise (HR_CET_/HR_peak_) as well as from the 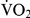
_CET_/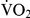
_peak_ ratio and from the 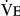
_CET_/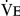
_peak_ ratio.

### Statistical Analyses

Results are reported as mean ± SD. The normality of the variables was checked by the Kolmogorov-Smirnoff test. Subjects were divided into tertiles of endurance time (Group 1: n = 32, endurance time <4 min; Group 2: n = 31, endurance time from 4 to 6 min; Group 3: n = 29, endurance time >6 min). The tertile cut-off points were chosen to reflect the desirable exercise duration for CET [25] and to balance the number of patients of the 3 groups. Between groups comparisons were assessed using ANOVA except for gender distribution which was analysed using chi-square tests. Possible relationships between endurance time during CET as the dependant variable, and FEV_1_, FVC, FEV_1/_FVC, TLC, FRC, RV, DLco, IC, delta IC from rest to end-exercise, delta SpO_2_ from rest to end-exercise, HR_peak_, W_peak_, 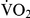
_peak_, 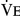
_peak_,
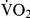
_VT_ as well as maximal voluntary contraction (MVC), the fall in Twq 10 minutes post exercise and dyspnea and leg fatigue at end of CET as independent variables, were assessed using Pearson's correlations. The possibility that a non-parametric model (Kendall tau rank correlation) would better describe the data than Pearson correlations was verified. Since the fit was not improved by the non-parametric model, only the linear model (Pearson's correlation) is reported. From the variables correlated to endurance time with p value <0.20, we first used general additive models to search for second and third-degree relations with endurance time. Secondly, stepwise regression analyses were used to determine independent predictors of endurance time during CET. Assumptions of linear regression were validated via linear relationship, normality and homoscedasticity. Multicollinearity was also ruled out to ensure validity of our analyses. A p value <0.05 was set as the statistical significance threshold. The analyses were performed with SAS version 9.2 (SAS Institute, Cary, NC).

## Results

### Subgroup characteristics

**Figure 1:**
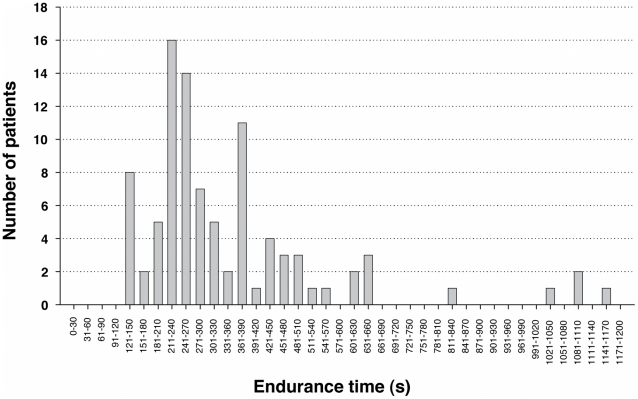
Range of endurance time (T_end_) during constant-workrate cycling exercise in all patients (n = 92). T_end_ varied from 120 to 1164 s.

The subject characteristics are reported in Table 2. No significant differences were found between groups for age, gender and body mass index. The range of T_end_ during CET is provided in Figure 1. The endurance time of the whole group of patients averaged 349 s, ranging from 120 to 1164 s.

**Table 2:** Subject characteristics and pulmonary function.

Variables	Group 1(n = 32)T_end_<4 min	Group 2(n = 31)T_end_ = 4–6 min	Group 3(n = 29)T_end_>6 min	ANOVAp value
**Age,** *years*	68±7	65±8	66±7	0.13
**Gender,** *M/F*	25/7	24/7	26/3	0.11
**BMI,** *kg/m^2^*	27.3±3.8	26.2±5.3	27.8±4.0	0.34
**FEV_1_,** *L*	1.14±0.42	1.29±0.46	1.42±0.40	0.54
**FEV_1_,** *% pred*	44±14	50±18	50±14	0.56
**FVC,** *L*	2.61±0.91	2.63±0.77	2.80±0.78	0.95
**FEV_1_/FVC,** *%*	44±9	49±10	51±6	0.11
**TLC,** *L*	6.62±1.29	6.57±1.38	7.44±1.07	0.11
**TLC,** *% pred*	108±14	109±15	118±17	0.23
**FRC,** *L*	4.18±0.89	4.47±1.28	5.13±1.03	0.36
**FRC,** *% pred*	123±22	135±32	148±31	0.83
**RV,** *L*	3.64±1.04	3.48±1.20	3.78±0 83	0.57
**RV,** *% pred*	135±38	135±47	142±36	0.62
**DLCO,** *mL/mmHg/min*	14.5±4.3	15.8±6.2	18.8±4.7	0.06
**DLCO,** *% pred*	46.2±11.9	52.1±18.0	58.6±14.3	0.15
**IC,** *L*	2.31±0.64	2.26±0.59	2.34±0.65	0.32
**IC,** *% pred*	86±17	86±19	85±18	0.39
**IC/TLC** *%*	33±9	33±9	31±6	0.69
**FRC/TLC** *%*	66±9	64±15	66±9	0.72
**RV/TLC** *%*	54±9	52±11	53±7	0.71
**Baseline SpO_2_,** %	96±3	97±2	97±2	0.50

Values are mean ± SD.

*Definitions of abbreviations: BMI: body mass index; FEV_1_: forced expiratory volume in 1 s; FVC: forced vital capacity; TLC: total lung capacity; FRC: functional residual capacity; RV: residual volume; DL_CO_: single-breath carbon monoxide diffusion capacity; IC: inspiratory capacity; SpO_2_: Oxygen pulse saturation.*

### Disease severity

Pulmonary function results were similar between the three groups (Table 2). No difference was found between groups in the fall in IC from rest to end exercise during CET (Table 3).

**Table 3:** Cardio-respiratory values at the end of incremental exercise and constant-workrate exercise test.

Variables	Group 1T_end_<4 min	Group 2T_end_ = 4–6 min	Group 3T_end_>6 min	ANOVAp value
***Incremental exercise***				
W_peak_ (Watt)	72±33* †	92±30	99±28	0.002
HR_peak_ (bpm)	130±18*	141±15	137±15	0.03
HR_peak_ (%pred)	85±11	91±8	89±10	0.07
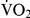 _peak_ (L/min)	1.07±0.36†	1.24±0.52	1.41±0.46	0.02
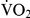 _peak_ (%pred)	60±17	74±34	74±22	0.08
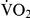 _VT_ (L/min)	0.79±0.25†	0.88±0.27	0.99±0.34	0.01
RER _peak_	1.08±0.11	1.13±0.09	1.11±0.08	0.16
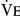 _peak_ (L/min)	43.5±12.3†	50.5±16.2	54.3±15.3	0.002
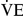 _peak_ (% MVV)	101±20	107±20	108±20	0.13
Dyspnea _peak_	7.1±2.0	6.6±2.3	7.7±2.0	0.15
Leg Fatigue _peak_	7.0±2.0	6.9±2.3	7.5±2.2	0.64
***Constant-workrate exercise test (CET)***				
HR_CET_ (bpm)	120±19* †	139±14	134±16	<0.0001
HR_CET_ (%peak)	92±10* †	99±8	98±9	0.01
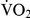 _CET_ (L/min)	0.96±0.36†	1.15±0.34	1.38±0.48	0.04
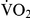 _CET_ (%peak)	94±21	99 ± 14	98±14	0.40
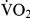 _CET_/ 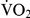 _VT_	126±27	137±23	140±29	0.13
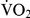 _CET_/ 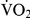 _peak_	94±21	99±14	98±14	0.41
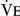 _CET_ (L/min)	39±11* †	48±13	54±17	0.0006
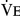 _CET_ (%peak)	93±10	97±8	99±14	0.14
ΔIC from rest (L)	0.65±0.41	0.47±0.40	0.45±0.41	0.15
ΔSpO_2_ from rest (%)	−3±4	−4±4	−2±2	0.18
Dyspnea _CET_	7.3±2.2	7.4±2.1	8.0±1.9	0.34
Leg Fatigue _CET_	6.2±2.5†	7.3±2.0	8.3±2.1	0.003
Fall in Twq after CET (% baseline)	17±21	22±15	19±24	0.65

Values are mean ± SD. Peak values were obtained at the end of incremental exercise. Constant-workrate exercise values were obtained at the end of constant-workrate exercise.

*p≤0.02 versus group 2.

† p≤0.03 versus group 3.

*Definitions of abbreviations: W_peak_: peak workrate capacity; HR: heart rate;* 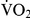 *: oxygen consumption;* 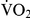 *_VT_: ventilatory threshold;* 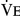 *: minute ventilation; MVV: maximal voluntary ventilation; RER: respiratory exchange ratio; CET; constant-workrate exercise test; IC: inspiratory capacity; Twq: Quadriceps twitch tension; SpO_2_: Oxygen pulse saturation.*

### Aerobic fitness and quadriceps function

**Figure 2:**
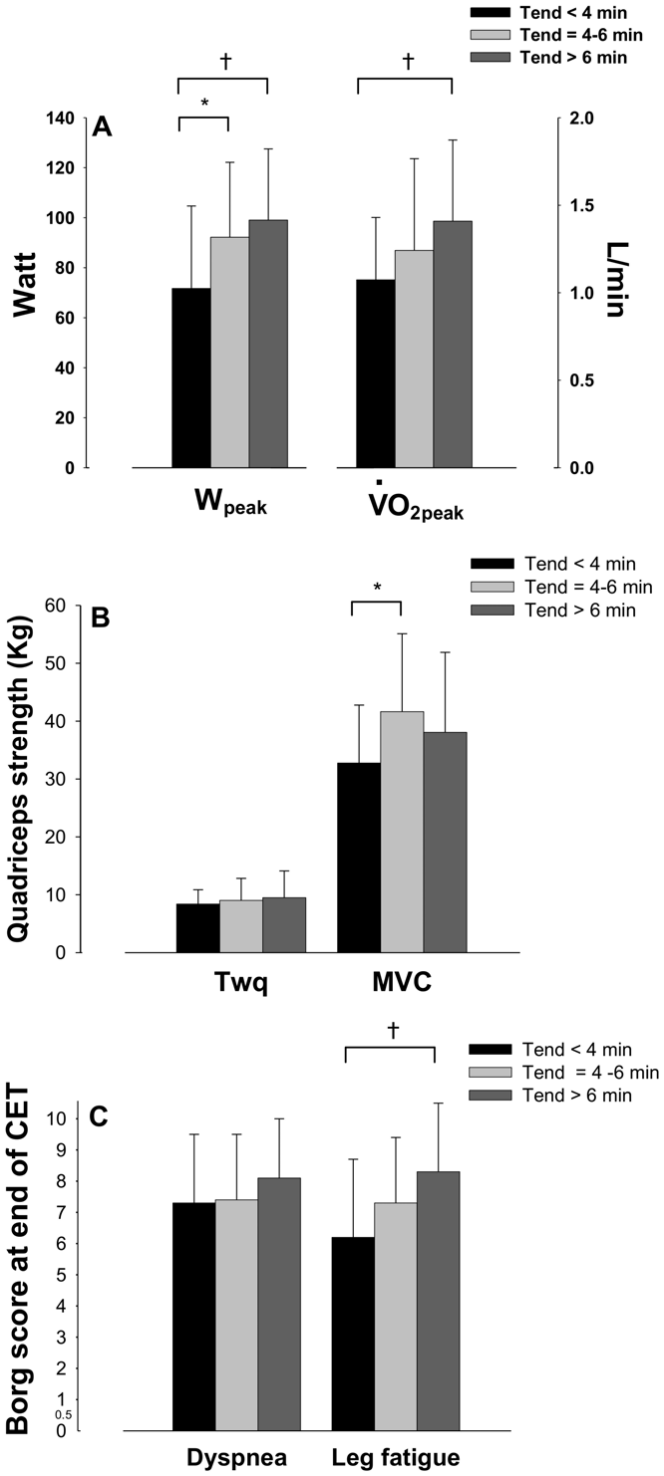
Selected correlates of endurance time. Group mean values for *i)* peak workrate (W_peak_) and peak oxygen consumption (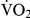
_peak_) during incremental exercise (panel A); *ii)* quadriceps strength assessed during magnetic stimulation of the femoral nerve (Twq) and maximal voluntary contraction (MVC) (panel B) and *iii)* symptom scores at the end of constant-workrate cycling exercise test (CET) (panel C) in group 1 (Tend<4 min, black bars), group 2 (Tend = 4–6 min, bright grey bars) and group 3 (Tend>6 min, dark grey bars) patients. * p≤0.02 versus group 2; † p≤0.01 versus group 3.

Patients in group 1 had lower aerobic fitness than group 2 and/or 3 when considering W_peak_, 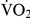
_peak_ (Figure 2a) and 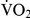
_VT_ (Table 3). MVC was lower in group 1 as compared with group 2 (Figure 2b). Twq (Figure 2b) and the fall in Twq after CET (Table 3) were not different across the three groups.

### Tolerance to dyspnea and leg fatigue

Group mean values for symptom scores at end of CET are shown on Figure 2c. There were no significant differences between groups in dyspnea at end of CET. However, leg fatigue scores at end of CET were lower in group 1 when compared with group 3.

### Technical factors

Cardio-respiratory values at the end of incremental exercise and CET are reported in Table 3. HR_peak_ expressed in % predicted value was not significantly different between groups. 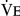/MVV at peak incremental exercise was above 100% in the 3 groups. Respiratory exchange ratio at the end of incremental exercise was also not different across the three groups. Overall, these results would suggest that patients of the 3 groups achieved a truly maximal performance during incremental testing. There was no significant difference between groups in dyspnea or leg fatigue at end of incremental exercise suggesting that patients in the three groups reached symptom limitation.

During constant exercise, HR_CET_ expressed in % of the peak value (reached during incremental exercise) was significantly lower in group 1 as compared with group 2 and 3 (Table 3). However, there was no significant difference in 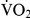
_CET_ and 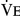
_CET_ between the three groups when these parameters were expressed in % peak value suggesting that the intensity of CET was similar between groups. Thus, shorter exercise duration in group 1 could not be explained by greater exercise intensity during CET.

**Figure 3:**
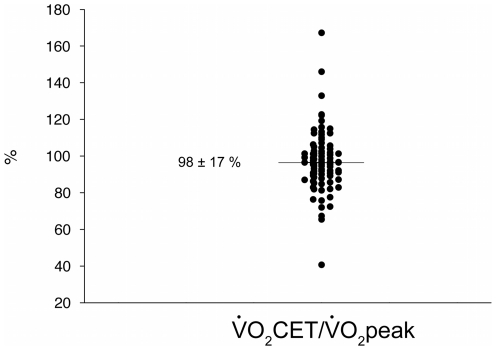
Actual exercise intensity during constant-workrate cycling exercise (CET) in all patients (n = 92). Data are expressed as the oxygen consumption at the end of CET to peak oxygen consumption during incremental exercise ratio (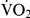
_CET_/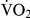
_peak_).

Despite the fact that CET was performed at a fixed proportion of W_peak_, there was a large interindividual variability in the actual exercise intensity during CET as assessed by the 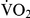
_CET_/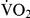
_peak_ ratio (Figure 3).

### Predictors of endurance time to CET

Correlation coefficients between independent variables and T_end_ are reported in Table 4. FEV_1_, FEV_1_/FVC, TLC, DL_CO_, IC, W_peak_, 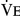
_peak_, 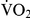
_ peak_, 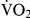
VT and leg fatigue score at the end of CET were all positively associated with endurance time in the univariate analysis. From these variables that significantly correlated with T_end_ in univariate analyses, only 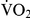
_VT_ and leg fatigue score at end of CET were independent predictors of endurance time variability in the stepwise regression analysis. This model explained only 25% of the variability in endurance time (cumulative r^2^ = 0.25, p<0.0001).

**Table 4:** Correlation analysis to endurance time.

Variables	r*	p
***Disease severity***		
FEV_1_ (L)	0.39	<0.0001
FEV_1_/FVC (%)	0.27	0.008
TLC (L)	0.24	0.03
FRC (L)	0.15	0.15
RV (L)	0.07	0.52
DL_CO_ (mL/mmHg/min)	0.36	0.002
IC (L)	0.24	0.01
ΔIC from rest to end-exercise (L)	0.12	0.29
ΔSpO_2_ from rest to end-exercise (%)	0.16	0.22
***Aerobic fitness and quadriceps function***		
W_peak_ (W)	0.38	0.0002
HR_peak_ (bpm)	0.13	0.19
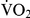 _peak_ (L/min)		
	0.40	<0.0001
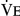 _peak_ (L/min)	0.43	<0.0001
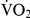 _VT_ (L/min)	0.40	0.0002
MVC (Kg)	0.22	0.05
Fall in Twq 10 min after CET (%Rest)	0.10	0.36
***Tolerance to dyspnea and leg fatigue***		
Dyspnea score at end of CET	0.14	0.18
Leg fatigue score at end of CET	0.33	0.001
***Technical factors***		
HR_peak_ (% predicted value)	0.13	0.20
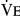 /MVV	0.17	0.10
HR_CET_/HR_peak_	0.08	0.41
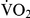 _CET_/ 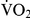 _peak_	0.05	0.63
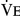 _CET_/ 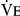 _peak_	0.02	0.79

Values are mean ± SD.

*r is the Pearson's correlation coefficient.

*Definitions of abbreviations: CET: constant-workrate exercise test; FEV_1_: forced expiratory volume in one second; FVC: forced vital capacity; TLC: total lung capacity; FRC: functional residual capacity; RV: residual volume; DL_CO_: single-breath carbon monoxyde diffusion capacity; IC: inspiratory capacity; SpO_2_: Oxygen pulse saturation; W_peak_: peak workrate capacity; HR: heart rate;* 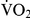
_peak:_ *peak oxygen consumption;* 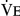 *: minute ventilation; MVC: maximal voluntary contraction; Twq: quadriceps twitch tension; MVV: maximal voluntary ventilation.*

## Discussion

Constant-workrate exercise testing was designed to assess exercise capacity at a fixed and predefined intensity across individuals. Herein we report that, in fact, this exercise testing modality was performed at highly variable relative intensities across individuals, resulting in large variations in the endurance time from one patient to another. The ability to tolerate leg fatigue during CET and the ventilatory threshold, as a reflection of aerobic fitness, were independent predictors of the variation in endurance time to CET. However, the ability of the physiological variables that were assessed in the present study to collectively explained the interindividual variability in endurance time to CET was modest, our regression model explaining only 25% of the variability in endurance time.

### Disease severity

Although we did not observed significant differences between groups in respiratory parameters, several pulmonary function indices (FEV_1_, FEV_1_/FVC and DL_CO_) positively correlated with endurance time (Table 4), suggesting that disease severity was associated with lower endurance time. These results corroborate to the well documented relation between ventilation limitation and exercise tolerance [26]. However, none of the parameters was independently associated with endurance time in the multiple regression analysis, suggesting that non-respiratory variables may be stronger determinants of endurance time variability than airflow limitation in COPD patients.

### Aerobic capacity and quadriceps function

As hypothesized, we found a lower peak exercise capacity (Figure 2a) and ventilatory threshold (Table 3) in group 1 than in group 2 and 3, suggesting that lower aerobic fitness was associated with reduced endurance time in patients with COPD. We also observed that quadriceps strength (Figure 2b) was lower in group 1 patients, suggesting that reduced quadriceps force was associated with shorter endurance time. Those variables all positively correlated with endurance time (Table 4).

### Tolerance to dyspnea and leg fatigue

On average, patients from the 3 groups reached similar peak effort perception as evidenced by the similar dyspnea and leg fatigue scores at the end of incremental exercise. However, patients in group 1 reached a lower leg fatigue score at end of CET than group 3 (Figure 2c). This is important since this variable correlated with endurance time during CET (Table 4). Furthermore, subjects from all groups presented similar ventilatory limitations as highlighted by similar end-CET dyspnea and 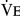/MVV levels. Consequently, lower leg fatigue score in group 1 patients may reflect that these individuals were less tolerant to leg fatigue during exercise. This statement is consistent with the notion that less fit individuals reach lower symptom scores during exercise compared to fitter individuals more accustomed to the uncomfortable sensations of exercise [24].

### Technical factors

We evaluated the possibility that an underestimation in peak workrate capacity may have lead to unduly prolonged CET. The fact that HR_peak_ expressed as % predicted value, 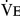
_peak_/MVV, and respiratory exchange ratio at the end of incremental exercise were not different between the 3 groups indicates that the ability to reach peak exercise capacity was comparable among them. Therefore this technical factor could not explain the variability in endurance time. A
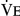
_peak_/MVV ratio >1 and a respiratory exchange ratio >1.1 in all three groups suggest that incremental exercise testing was truly maximal [27]. Also, higher exercise intensity during CET could not be responsible for the shorter exercise duration in group 1, since 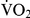
_CET_ expressed as % peak value reached during incremental exercise and 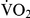
_CET_/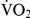
_VT_ ratio tended to be lower in these patients (Table 3).

Although relative exercise intensity during constant-workrate exercise was *a priori* identical in all subjects (80% W_peak_), we observed a large interindividual variability in 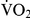
_CET_/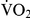
_peak_ ratio (Figure 3) indicating that selecting exercise intensity based on a fixed ratio of workload does not result in the same physiological stress.

We took advantage of this dataset to take a clinical and pragmatic approach to try to understand the mechanisms explaining the observed variations in endurance time. One strength of the present study is that all patients were evaluated in the same exercise laboratory under standardized exercise procedures provided by experienced investigators. The sample size is relatively large and physiological variables covering various aspects of exercise physiology were assessed. We acknowledge that the tercile analysis may have suffered from a reduced power to detect some relevant differences between the three categories of endurance time. This potential limitation was addressed by also performing correlation analyses from the whole sample size. These two approaches could be viewed as complementary in the identification of predictors of the variability in endurance time. We appreciate the retrospective nature of this study and the fact that investigating the variability in exercise duration was not the primary reason for the exercise testing. In this perspective, we could not extend our analysis to some other physiological (i.e maximal respiratory pressures) and non-physiological (i.e. anxiety, depression) factors potentially influencing endurance time in COPD. Finally, giving the majority of men in our cohort, caution should be taken before applying the findings to women in COPD.

Importantly, this retrospective study was not designed to determine how to select CET intensity and homogenize exercise duration, but to elucidate potential factors responsible for the high variability in endurance time. Still, one possible approach could be to use different relative exercise intensities according to disease severity and aerobic capacity. Whether this approach would be successful is difficult to predict because only a modest fraction of the variability in endurance time could be predicted from our multiple regression model. The clinical message emerging from this study is that predicting the endurance time during contant-workrate cycling exercise from clinical and physiological parameters is likely to be difficult. A physiological approach based on the determination of the power-duration relationship is impractical in the clinical setting. A more realistic method could be to realize a familiarization constant-workrate cycling exercise test at a given intensity (e.g. 80%) and, if necessary, to adjust the intensity in subsequent tests in order to obtain the desire exercise duration [25].

### Conclusion

Although worse pulmonary function was related to short endurance time, 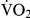
VT and leg fatigue Borg score were the only independent predictors of endurance time variability in this study. Yet, taken together, the variables assessed in the present investigation only accounted for 25% of endurance time variability during CET, suggesting that a large fraction in endurance time could not be explained by those physiological parameters. This study highlights the complex nature of exercise limitation in COPD. Furthermore, exercise tests performed at a fixed proportion of peak incremental workload capacity do not provide the same physiological stress among individuals. This approach is unlikely to result in the optimal 4–7 min duration for CET. Further studies are needed to learn how to individualize the exercise intensity during CET in COPD patients.
